# Annotations

**Published:** 1906-08-18

**Authors:** 


### ANNOTATIONS.

### Out-Patients Again.

An incidental reference in the report of the Hospital Sunday Fund meeting brings up once more the burden of out-patients. Dr. Glover expressed his concern at finding that 571,666 out-patient attendances were registered at the London Hospital during the year. Mr. Sydney Holland justified the numbers on the ground that the London Hospital had only one other institution to help it in dealing with the whole of East London, and the Distribution Committee awarded to the London Hospital nearly one-tenth of the whole available sum. We do not question the justice of the committee, for it is probable that any strictures applicable to the methods of investigation in vogue there would apply with equal force to any other hospital which deals in hundreds of thousands, but the massive figures supply their own argument. If we set off the odd 70,000 against the Sundays of the year there remains an average attendance of rather over 1,500 for every week day. In view of this rough calculation it is not necessary to labour the impossibility of efficient investigation into the circumstances of the immense body of applicants, and although Mr. Sydney Holland is satisfied that there is no abuse, there are probably many who will find themselves unable to share his satisfaction. The truth is that at present the hospital out-patient system puts a premium on dishonesty, for the word of the applicant, and the character of his clothing remain the only criteria of his fitness for the charity he seeks. The wise rogue, therefore, can hardly be exposed. But the hospitals are not to blame, for they are not staffed for extended inquiries. It is hard to see what solution awaits this problem unless it be the establishment by the State of a charity organisation department whose sole business shall be the prevention of abuses..

### The Influence of Bitters on Digestion.

Badly cooked food, hurried meals, and imperfect mastication are potent factors in producing the dyspepsia which is so common a complaint amongst the thousands of men and women who daily go up to town and return home in the evening. The medical out-patient rooms of the hospitals are crowded with patients who seek relief for their indigestion, and who derive the greatest benefit from some simple mixture containing gentian or calumba as one of its active ingredients. Such substances are comparatively harmless. They act reflexly to produce an increased flow of gastric juice through the sensory nerves of the mouth and palate. Experiment has shown that when gentian, calumba, or quassia is introduced directly into the stomach it produces no greater result than would be produced by the ingestion of a similar quantity of distilled water. But when the mouth and tongue are wetted with the bitter infusion, although no extra flow of gastric juice has resulted, yet, if food is given immedately afterwards, a very considerable increase in the flow of gastric juice is invariably noticed as soon as the food reaches the stomach. There is no doubt, therefore, that the action of bitters is essentially due to their influence upon the sensory nerves of the mouth, and not to their action upon the gastric mucous membrane. Such bitters, therefore, should always be given in a fluid form just before a meal if the best results are to be obtained. But there exists a dangerous class of remedies known as "pick-me-ups," "tonics," and the like, nearly all of which depend for their action upon alcohol combined with strychnia, nux vomica, or bromides. Powerful drugs like alcohol, strychnia, and the bromides cannot be taken with impunity by people whose education does not allow them to gauge their action except in the roughest manner by their own sensations. It would be more rational to seek the cause of the dyspeptic symptoms, to insist upon better cooked food, to have the teeth overhauled by a good dentist, and to spend a little more time over eating. School managers should provide the future wife of artisans with instruction in cookery, and thereby do something to save the pockets of husbands and sons, and protect them from the curse of drink, for a well-fed man free from dyspepsia is rarely a drunkard.

### Nasal Irritation and Meddlesome Midwifery.

Sir Lauder Brunton used to tell his students how a highly qualified young practitioner once wearied out the patience of the friends of the lying-in woman at his first case by waiting too long for the placenta. They surreptitiously sent for the country quack. Assuming an air of much importance when he came into the sick chamber he asked the M.D. (Lond.) if he had "quilled her." Not knowing what this meant the young doctor admitted he had not done so. Thereupon the quack took his snuff-box from his vest-pocket and, having filled a crow quill with the snuff, blew it up the patient's nostril and caused a violent fit of sneezing, during which the obdurate placenta was expelled, and the quack departed, chuckling at the discomfiture of the grand learned young doctor. Two daring and imaginative investigators, Drs. Jerusalem and Falkner have recently found that "in many cases" the spontaneous contractions of the uterus after the birth of the child, were unable to expel the placenta, until the nose, and especially the anterior ends (sic.) of the inferior turbinal bone, or the tuberculum septi, had been touched with cotton wool dipped in a 10 per cent. solution of cocaine. The investigators seemed to have been easily satisfied. Hitherto the rhinologists and the gynæcologists, like the British and the Swiss, and for much the same reasons, have been very good friends, but have these days of peace passed away? That the rhinologists can lawfully look upon the uterus, during the interesting time of parturition, as within their sphere of influence, will be welcome news to many struggling nose-specialists, and during the period which elapses between the birth of the child and the expulsion of the placenta it may be as well to paint the turbinates with cocaine as to do anything else. It can scarcely be called "meddlesome midwifery," and still it is "doing something."

